# Isolation and identification of *Fusarium* species from the water systems of ICUs and transplant wards of hospitals and determination of the *in vitro* susceptibilities of isolates to conventional antifungals

**DOI:** 10.3389/ffunb.2025.1564237

**Published:** 2025-05-14

**Authors:** Fatemeh Mirhasani, Roshanak Daie-Ghazvini, Seyed Jamal Hashemi, Sadegh Khodavaisy, Pegah Ardi, Zahra Rafat, Davoud Roostaei, Heidar Bakhshi, Fatemeh Amirzadeh-Ghasemi

**Affiliations:** ^1^ Department of Medical Parasitology and Mycology, School of Public Health, Tehran University of Medical Sciences, Tehran, Iran; ^2^ Department of Medical Parasitology and Mycology, School of Medicine, Guilan University of Medical Sciences, Rasht, Iran; ^3^ Department of Pharmacology, School of Medicine, Guilan University of Medical Sciences, Rasht, Iran

**Keywords:** *Fusarium oxysporum* complex, *Fusarium fujikuroi* complex, water system, hospital, antifungal susceptibility testing

## Abstract

**Background:**

The rising prevalence of nosocomial infections, particularly those caused by airborne fungal spores in operating rooms and intensive care units (ICUs), has become a significant public health concern. *Fusarium* species in water systems pose a severe threat to immunocompromised patients and can disseminate as aerosols through devices such as faucets and showers. This study aimed to isolate and identify *Fusarium* species from the water systems of the ICUs and transplant units at Imam Khomeini Hospital Complex and Shariati Hospital in Tehran, Iran, as potential sources of future outbreaks. Additionally, the study sought to determine the *in vitro* susceptibilities of the isolates to conventional antifungal agents.

**Methods:**

Sterile swabs and open plates containing Sabouraud dextrose agar (SDA) with chloramphenicol were used to collect water samples from sink surfaces, shower trays, faucets, and around the drains of sinks, as well as from bathroom areas. Swab samples were cultured, and the open-plate samples were evaluated for the growth of *Fusarium* species. The validation of all *Fusarium* sp. isolates was performed using DNA sequencing of the translation elongation factor 1-alpha (TEF-1α) gene. The antifungal susceptibility patterns of each isolate were tested against voriconazole, itraconazole, posaconazole, caspofungin, and amphotericin B using the Clinical and Laboratory Standards Institute (CLSI) broth microdilution method for filamentous fungi.

**Results:**

*Fusarium* species were recovered from six out of 362 water system samples, representing 1.65% of the total. Five isolates were identified as *Fusarium oxysporum* from the *F. oxysporum* complex, while one isolate was identified as *Fusarium proliferatum* from the *Fusarium fujikuroi* complex. All isolates were obtained from sinks (three isolates) and faucets (three isolates) at Imam Khomeini Hospital. Antifungal susceptibility testing revealed that posaconazole, voriconazole, and amphotericin B were the most effective drugs against all *Fusarium* isolates, with no instances of resistance to these antifungal agents observed. However, non-wild-type isolates were noted for the other drugs tested.

**Discussion:**

The isolation of pathogenic *Fusarium* species from water samples collected in the ICU wards of the Imam Khomeini Hospital Complex underscores the urgent need to implement effective control and prevention measures in hospital water systems.

## Introduction

The genus *Fusarium* consists of hyaline, septate mycelium typically found in plants, soil, and various organic substrates (Ekwomadu and Mwanza, 2023). In recent years, these fungi have emerged as the second most common cause of invasive fungal infections among immunocompromised patients (Nucci and Anaissie, 2023). *Fusarium* species have the potential to persist in water systems, including those in hospitals, for extended periods (Hino et al., 2020). Some studies have indicated that nosocomial infections can be attributed to *Fusarium* species (Balmas et al., 2021; Douglas et al., 2023). The prevailing hypothesis suggests that *Fusarium* species present in water become aerosolized when the water flows through fixtures, such as taps and showers, and are subsequently inhaled by immunocompromised patients who are at an elevated risk for fusariosis (Balmas et al., 2021).

Over 300 species of *Fusarium* have been identified, with approximately 70 of these species implicated in human infections (Cighir et al., 2023). The species most frequently associated with fusariosis are *Fusarium solani* (60%) and *Fusarium oxysporum* (20%) (Kausar et al., 2021). These two species have been classified as species complexes, and several new species have been identified and described recently. The species complexes frequently associated with human infections include the *F. solani* species complex (FSSC), *F. oxysporum* species complex (FOSC), *Fusarium tricinctum* species complex (FTSC), *Fusarium incarnatum-equiseti* species complex (FIESC), *Fusarium fujikuroi* species complex (FFSC), *Fusarium dimerum* species complex (FDSC), and *Fusarium chlamydosporum* species complex (FCSC) (Abdurakhmonov, 2024).

*Fusarium* infections primarily present as keratitis and onychomycosis in immunocompetent individuals, while severe disseminated infections are observed in those with compromised immune systems (Nucci and Anaissie, 2023). Fungemia in patients with significant immunosuppression leads to localized necrosis following secondary dissemination to the skin (Varghese et al., 2023). Multiresistance to antifungal agents is a characteristic feature of all *Fusarium* species, making these fungi particularly challenging to manage therapeutically (Wiederhold, 2020). Each species exhibits a unique resistance pattern to specific antifungal agents. Furthermore, *Fusarium* species lack a standardized minimum inhibitory concentration (MIC), complicating the prediction of antifungal susceptibility for individual strains (Wiederhold, 2020). Antifungal susceptibility testing (AFST) should be integrated into standard patient management practices to enhance therapeutic effectiveness, especially in cases of severe infections. To the best of our knowledge, there is limited information regarding the prevalence of *Fusarium* spp. in hospital water systems, which may serve as potential sources for future outbreaks, as well as the *in vitro* susceptibilities of isolates to antifungals. This scarcity of data is primarily due to the fact that most laboratories do not routinely perform AFST, combined with the difficulties many laboratories face in accurately identifying *Fusarium* species. Given the hypothesis that *Fusarium* species present in water become aerosolized when the water flows through fixtures, subsequently being inhaled by immunocompromised patients at elevated risk for fusariosis, the present study aimed to i) evaluate the prevalence of *Fusarium* spp. in the water systems of the intensive care units (ICUs) and transplant wards of Imam Khomeini Hospital Complex and Shariati Hospital in Tehran and ii) investigate AFST of the strains to assist clinicians in making optimal antifungal decisions.

## Materials and methods

### Ethics statement

Ethical approval for this study was obtained from the Ethics Committee of Tehran University of Medical Sciences (IR.TUMS.SPH.REC.1402.006).

### Sampling, isolation, and growth conditions

This descriptive cross-sectional study was conducted over a period of 19 months (from May 2022 to December 2023) in the transplant wards and ICUs of Imam Khomeini Hospital Complex and Shariati Hospital in Tehran, the capital and largest city of Iran. During the study period, six sterile cotton-tipped applicators were used to collect water samples from the surfaces, shower trays, faucets, and around the sinks with a circular and one-way motion of the swabs (Anaissie et al., 2001). Additionally, to collect water samples from the bathrooms, sampling was performed with the water running for 35 minutes. In this setup, plates containing Sabouraud dextrose agar (SDA) with chloramphenicol (SC, Merck, Germany) culture medium were placed at a height of 1.5 m above the ground, next to the shower, with the plate lid cracked open for 15 minutes. The collected samples were then transferred to the Medical Mycology Laboratory of Tehran University of Medical Sciences (TUMS) for mycological analysis. The SC plates were incubated at 25°C for 72 hours and examined daily for growth of *Fusarium* spp. The swabs were also cultured on SDA medium and incubated at a temperature of 25°C for 72 hours. The purified colonies were then cultured on subsequently Potato Dextrose Agar medium (PDA; Merck, Germany) for 5–7 days at 25°C. Five to seven *Fusarium* spp. at 25°C were further identified by colony morphology and lactophenol cotton blue (LPCB) mounts.

*Fusarium* colonies typically appear fast-growing, with velvety to cottony texture, and can range in color from white to yellow, pink, red, or purple depending on the species; microscopically, *Fusarium* is characterized by the production of both macroconidia (large, sickle-shaped) and microconidia (smaller, often one-celled spores) (Chidambaram et al., 2017).

### Molecular techniques

The validation of all *Fusarium* spp. isolates was carried out using DNA sequencing of the translation elongation factor 1-alpha (TEF-1α) gene. The genomic DNA was extracted from harvested colonies using the phenol–chloroform method. The amplification of the TEF-1α was performed with a pair of primers, EF1 (5′ ATGGGTAAGGAAGGACAAG 3′) and EF 2 (5′ GGAGAGTACCAGTGCATCAT 3′). Each polymerase chain reaction (PCR) contained 5 μL of 10× reaction buffer, 1.5 μL MgCl, 0.5 μL dNTP mixture, 0.25 μL Taq polymerase (Prime Taq, Genet Bio, South Korea), 0.5 μL of each primer, 1 μL of DNA template solution, and enough distilled water to reach a final volume of 50 μL. PCR was conducted with the following conditions: an initial denaturation step at 95°C for 5 minutes, 35 amplification cycles with denaturation at 94°C for 45 s, primer annealing at 55°C for 1 minute, and elongation at 72°C for 1 minute, followed by a final elongation step at 72°C for 6 minutes. Positive PCR products were examined by staining with DNA safe stain (4 µL) and electrophoresis on 1.5% agarose gel. The PCR products were subjected to sequencing and analyzed using the ChromasPro-Version 2.1.3 software. The EF sequences were compared with those available in the National Center for Biotechnology Information (NCBI; http://www.ncbi.nlm.gov/) genetic database using the Basic Local Alignment Tool (BLAST) algorithm and using only sequence identity values above 99%.

### Antifungal susceptibility testing

The Clinical and Laboratory Standards Institute document M38-A2 (CLSI M38-A2) broth microdilution protocol was used as a guideline for AFST of filamentous fungi (Espinel-Ingroff et al., 2009). The susceptibility of the *Fusarium* spp. isolates to voriconazole (VOR), posaconazole (PSZ), caspofungin (CAS), itraconazole (ITR), and amphotericin B (AMB) (all from Pfizer, Groton, CT, USA) was tested. The following dilutions were developed: 0.06–64 µg/mL for VOR, 0.06–64 µg/mL for ITR, 0.06–64 µg/mL for PSZ, 0.008–8 µg/mL for CAS, and 0.06–64 µg/mL for AMB. A suspension equivalent to 0.5 McFarland was prepared from the *Fusarium* culture on PDA by suspending the fungal cells in 6 mL of sterile distilled water, and the concentration was spectrophotometrically adjusted according to CLSI M38-A2. Subsequently, 100 µL of fungal inocula was dispensed into wells of microplates, and the results were read after 48 hours of incubation at 35°C. The quality reference strains of *Candida parapsilosis* (ATCC 22019), *Candida krusei* (ATCC 6258), and *Aspergillus flavus* (ATCC 2004304) were used as CLSI control strains.

### Statistical analysis

Data were analyzed using the Excel software with descriptive and analytical statistics. Student’s *t*-test was used to compare the means between the two groups with a 95% confidence interval and *p* < 0.05 significance level.

## Results

During a period of 19 months, a total of 362 water samples were collected, including 144 samples from Imam Khomeini Hospital Complex and 218 samples from Shariati Hospital. Of these, 32 samples were collected using open plates and 330 samples using sterile swabs. After culture, the colony appearance of six samples (1.65%) showed white, cottony colonies with abundant aerial mycelia of *Fusarium* spp. Five isolates were identified as *F. oxysporum* from the *F. oxysporum* complex, and one isolate was identified as *Fusarium proliferatum* from the *F. fujikuroi* complex.

All isolates were obtained from sinks (three isolates) and faucets (three isolates) at the Imam Khomeini Hospital Complex. Three isolates were obtained from the general ICU: two from the cardiac ICU (CICU) and one from the thorax and lung transplant ICU (TLTICU) (**Table 1**).

**Table 1:** Detailed information related to six *Fusarium* species isolated from the water systems in Imam Khomeini Hospital Complex.

Isolate number	Isolate	Species complex	ward	Sampling location
**1**	*Fusarium oxysporum*	FOSC	General ICU	Sink
**2**	*F. oxysporum*	FOSC	General ICU	Faucet
**3**	*F. oxysporum*	FOSC	General ICU	Faucet
**4**	*F. oxysporum*	FOSC	TLTICU	Faucet
**5**	*Fusarium proliferatum*	FFSC	CICU	Sink
**6**	*F. oxysporum*	FOSC	CICU	Sink

FOSC, *F. oxysporum* species complex; FFSC, *Fusarium fujikuroi* species complex; TLTICU, thorax and lung transplant intensive care unit; CICU, cardiac ICU.

The results of AFST are presented in **Table 2**. We accessed the susceptibility of six *Fusarium* species isolates to five antifungal drugs (voriconazole, itraconazole, posaconazole, caspofungin, and amphotericin B) by the epidemiological cutoff values (ECVs) of antifungal agents determined by Espinel-Ingroff et al (WHO (world health organization), 2022).

**Table 2:** *In vitro* antifungal susceptibility pattern of *Fusarium* species isolated from the water systems in Imam Khomeini Hospital Complex.

Antifungal drug	MIC/MEC range (μg/mL)	MIC/MEC (μg/mL)/sensitivity pattern					
		Isolate 1	Isolate 2	Isolate 3	Isolate 4	Isolate 5	Isolate 6
Amphotericin B	2–4	2/W-T	4/W-T	2/W-T	2/W-T	2/W-T	2/W-T
Itraconazole	-	>32/N-W-T	>32/N-W-T	>32/N-W-T	>32/N-W-T	>32/N-W-T	>32/N-W-T
Voriconazole	1–4	2/W-T	4/W-T	2/W-T	2/W-T	1/W-T	2/W-T
Posaconazole	0.5–1	1/W-T	0.5/W-T	0.5/W-T	1/W-T	1/W-T	0.5/W-T
Caspofungin	-	>8/N-W-T	>8/N-W-T	>8/N-W-T	>8/N-W-T	>8/N-W-T	>8/N-W-T

MIC, minimum inhibitory concentration; MEC, minimum effective concentration; W-T, wild-type; N-W-T, non-wild-type.

All isolates were wild-type for amphotericin B, posaconazole, and voriconazole, while non-wild-type isolates were observed for the other drugs. AFST showed that posaconazole (MIC range, 0.5–1 μg/mL), voriconazole (MIC range, 1–4 μg/mL), and amphotericin B (MIC range, 2–4 μg/mL) were the most active drugs against all *Fusarium* isolates, and no case of resistance to these antifungal agents was observed, while non-wild-type isolates were observed for the other drugs.

## Discussion

Recent advancements in the treatment of patients admitted to ICUs or those who have undergone organ transplantation have not diminished the severity of fusariosis, and this infection remains a notable contributor to mortality in these patients (Ekwomadu and Mwanza, 2023). The World Health Organization categorized the genus *Fusarium* as part of the “high priority group” of pathogens that pose significant public health risks and outlined it in the fungal priority pathogens list (FPPL) (WHO (world health organization), 2022). *Fusarium* spp. may inhabit water distribution systems, including those of hospitals, and can persist in the water system for years (Sautour et al., 2012). The water system has the potential to spread these organisms through aerosols generated by showers and sinks, and the isolates obtained from the water system may also lead to nosocomial infections (Anaissie et al., 2001).

In the present study, five *F. oxysporum* isolates and one *F. proliferatum* isolate were recovered from six (1.65%) water system samples. Anaissie et al., for the first time, demonstrated that hospital water systems can be reservoirs for opportunistic fungi, particularly *F. oxysporum* and *F. solani* (Anaissie et al., 2001). Numerous reports published in the literature have confirmed the diversity of situations regarding the recovery rates of *Fusarium* spp. from hospital water distribution systems. No strains of *Fusarium* were identified in the hospital water systems located in Nijmegen, Netherlands (Warris et al., 2003), nor Athens and Thessaloniki, Greece (Panagopoulou et al., 2002). Furthermore, it was observed that less than 1% of samples tested positive at a hospital located in Thessaloniki, Greece (Arvanitidou et al., 1999). Conversely, molds belonging to the genus *Fusarium* were detected in 57% of water samples collected from a hospital situated in Houston, USA (Anaissie et al., 2001).

Also, in the current investigation, all isolates were obtained from sinks and faucets at Imam Khomeini Hospital Complex, the largest hospital in the Middle East. Considering the fact that there are many old buildings in this hospital, the construction and renovation of different buildings of this hospital can be an important source for *Fusarium* conidia (Sautour et al., 2012). We hypothesize that variations in hospital infrastructure, maintenance practices, and water quality may have contributed to the significant difference in isolate recovery. For instance, the water management protocols and the sanitation practices in place at each hospital could impact the presence of airborne fungal spores. Therefore, we point out the need for further studies that include a comparison of these factors across institutions to better understand their influence on the prevalence of *Fusarium* species. Additionally, there is the possibility of sampling bias. The specific locations sampled (sinks, faucets, and showers) may not be uniformly contaminated across both hospitals or may have been affected by variable water flow and usage patterns. Future research could benefit from a randomized sampling strategy and an extended duration to capture temporal variations in fungal presence more comprehensively.

Also, the presence of biofilms in the sinks and faucets could be another source of water contamination in the present study (Sautour et al., 2012). In accordance with the present investigation, in a previous study, the sink was reported to be the most isolated place of *Fusarium* spp., and from 92 sink drains tested, 72 (88%) yielded contamination with *Fusarium* spp (Sautour et al., 2012).

*Fusarium* species are commonly resistant to many antifungal drugs currently available in clinical settings. The intrinsic resistance of *Fusarium* spp. to azoles, along with high MICs/minimum effective concentrations (MECs) to polyenes and echinocandins, has been reported, although some studies have reported successful treatment with antifungals (Sav et al., 2018).

In the present study, the *in vitro* activity of five common antifungal drugs including voriconazole, itraconazole, posaconazole, amphotericin B, and caspofungin against *Fusarium* isolates was investigated. Posaconazole showed greater *in vitro* activity than other tested antifungal drugs against the *Fusarium* isolates. Therefore, the results showed its superiority to amphotericin B as the primary therapy of invasive fusariosis.

In a study conducted by Paphitou et al (Paphitou et al., 2002), the sensitivity of various *Fusarium* species (n = 22) against five antifungal drugs (voriconazole, fluconazole, amphotericin B, posaconazole, and ravuconazole) was investigated. According to their findings, posaconazole and voriconazole exhibited promising antifungal activities, which is completely consistent with the findings of the present study.

Therefore, in severely immunocompromised patients, it is imperative to take all necessary precautions to minimize their exposure to *Fusarium* spp. This may include putting high-risk patients in rooms equipped with High-Efficiency Particulate Air (HEPA) filters and maintaining positive pressure, avoiding contact with potential sources of *Fusarium* spp., such as tap water, and ensuring that showers are thoroughly cleaned before use by high-risk patients (Nucci and Anaissie, 2007).

The results of the present study showed that among the *Fusarium* spp. isolated from water samples, five isolates were identified as *F. oxysporum* from the *F.* oxysporum complex, and one isolate was identified as *F. proliferatum* from the *F. fujikuroi* complex. Additionally, it will be useful to conduct future studies to show how the picture will change after the necessary protective measures. Also, it is recommended to pursue further studies with an expanded sample size to validate our findings and enhance the applicability of our results.

Furthermore, the present study identified environmental isolates of *Fusarium* species, but it did not investigate their direct association with patient infections. This is a significant limitation of our research that we must emphasize. Therefore, further research is essential to establish the clinical relevance of these environmental isolates through a comprehensive epidemiological approach, possibly involving patient screening and clinical data correlation.

## Conclusion

The isolation of pathogenic *Fusarium* spp. in water samples gathered from ICU wards of the Imam Khomeini Hospital Complex highlights the necessity of implementing appropriate control and prevention methods in hospital water systems to reduce the infection risks associated with these potentially dangerous microorganisms.

The presence of *Fusarium* species in ICUs can indeed be complex. While *Fusarium* infections are often considered less common than those caused by other fungi, such as *Aspergillus*, this perception may be influenced by several factors:

1. The methodology used in culture and identification can significantly affect the reported prevalence of *Fusarium*. Traditional culture techniques may not effectively capture all fungal species present in the environment, especially if their growth conditions differ from those preferred by *Aspergillus* or if they require specific media for optimal growth. Molecular methods, such as PCR, can improve detection rates of *Fusarium* and other fungi that are underreported with standard culturing techniques.
2. *Fusarium* species are widely distributed in soil and plants, and they can be found in healthcare settings, especially in environments with plant material or organic matter. However, their actual counts in ICU air and surfaces compared to those of other fungi like *Aspergillus* may vary based on local environmental conditions, cleaning practices, and the specific design of the ICU.
3. Research indicates that *Aspergillus* is more commonly associated with invasive fungal infections in immunocompromised patients, particularly in hospital settings. In contrast, while *Fusarium* can cause serious infections, particularly in patients with weakened immune systems, it is considered less prevalent overall in clinical settings, although reports of fusariosis are increasing, and studying its presence can provide insights into potential sources of infection and transmission routes within ICUs and other high-risk areas. By focusing on *Fusarium*, researchers can contribute valuable knowledge to the field, helping to ensure early detection, appropriate treatment, and effective prevention strategies against this and other fungal pathogens.

## Data Availability

The datasets presented in this study can be found in online repositories. The names of the repository/repositories and accession number(s) can be found in the article/supplementary material.
